# Clinical and Functional Outcomes of Peri-Implant Fractures Associated with Short Proximal Femur Nails: Prevention Strategies and Key Insights

**DOI:** 10.3390/jcm14010261

**Published:** 2025-01-05

**Authors:** Ignacio Aguado-Maestro, Sergio Valle-López, Clarisa Simón-Pérez, Emilio-Javier Frutos-Reoyo, Ignacio García-Cepeda, Inés de Blas-Sanz, Ana-Elena Sanz-Peñas, Jesús Diez-Rodríguez, Juan-Pedro Mencía-González, Carlos Sanz-Posadas

**Affiliations:** 1 Department of Orthopaedic Surgery and Traumatology, Río Hortega University Hospital, C Dulzaina 2, 47012 Valladolid, Spain; iaguadom@saludcastillayleon.es (I.A.-M.); icepeda@saludcastillayleon.es (I.G.-C.); ideblassanzd@saludcastillayleon.es (I.d.B.-S.); asanzpe@saludcastillayleon.es (A.-E.S.-P.); jdiezrod@saludcastillayleon.es (J.D.-R.); 2 Discipline of Orthopaedics, Faculty of Medicine, University of Valladolid, Av Ramón y Cajal 7, 47007 Valladolid, Spain; csimon@saludcastillayleon.es (C.S.-P.); mencia_jua@gva.es (J.-P.M.-G.); 3 Department of Rehabilitation and Physical Medicine, Río Hortega University Hospital, C Dulzaina 2, 47012 Valladolid, Spain; efrutosr@saludcastillayleon.es; 4 Discipline of Physiotherapy, Faculty of Health Sciences, European University Miguel de Cervantes, C del Padre Julio Chevalier 2, 47012 Valladolid, Spain; sanzposadascarlos@gmail.com

**Keywords:** proximal femur fracture, short femur nail, peri-implant fracture, fragility fracture

## Abstract

**Background**: Hip fractures are prevalent among the elderly and impose a significant burden on healthcare systems due to the associated high morbidity and costs. The increasing use of intramedullary nails for hip fracture fixation has inadvertently introduced risks; these implants can alter bone elasticity and create stress concentrations, leading to peri-implant fractures. The aim of this study is to investigate the outcomes of peri-implant hip fractures, evaluate the potential causes of such fractures, determine the type of treatment provided, assess the outcomes of said treatments, and establish possible improvement strategies. **Methods**: We conducted a retrospective observational study on 33 patients with peri-implant hip fractures (PIFs) who underwent surgical management at Río Hortega University Hospital from 2010 to 2022. The collected data included demographics, initial fracture characteristics, the peri-implant fracture classification, implant details, surgical outcomes, functional scores, and complications. Functional capacity was evaluated using the Parker Mobility Score (PMS). **Results**: The cohort (91% female, mean age 87.6 years) included 34 peri-implant fractures. The mean time from the initial fracture to the PIF was 47.2 months (nine patients developed PIFs within 2 months). Most fractures (76%) were managed with implant removal and the insertion of a long intramedullary nail, with cement augmentation in 31% of cases. The mean surgical time was 102 min, and the average hospital stay was 9.6 days. Postoperative complications occurred in 27%, with a perioperative mortality rate of 9%. Functional capacity showed a significant decline, with an average PMS loss of 4.16 points. Mortality at one year post-PIF was 36%, rising to 83% at five years. Radiographic consolidation was observed in 72% of cases at an average of 6.04 months, though 24% of patients died before consolidation. Statistically significant correlations were found for PMS pre-index fracture (PMS1: r = 0.354, *p* < 0.05), pre-PIF (PMS2: r = 0.647, *p* < 0.001), and post-PIF (PMS3: r = 0.604, *p* < 0.001). **Conclusions**: Peri-implant hip fractures present complex challenges due to their surgical difficulty and impact on patient mobility and survival. Successful management requires individualized treatment based on fracture type, implant positioning, and patient factors. These findings underscore the need for preventive measures, particularly in implant choice and techniques like overlapping and interlocking constructs, to minimize the secondary fracture risk.

## 1. Introduction

Hip fractures represent a significant economic, social, and healthcare burden due to their high incidence in the elderly population over 65 years old. In Spain, approximately 40,000 hip fractures occur annually, with an incidence of about 500 cases per 100,000 inhabitants, and the healthcare costs of these fractures amount to 2.5 billion euros annually, with a total of 7218 quality-adjusted life years lost each year [1]. The average age of onset is 81.4 years [2], and mortality rates range from 5 to 10% at one month and from 20 to 30% at one year post-fracture. Of the survivors, half become dependent, and 10–20% move to nursing homes. However, the specific challenges associated with peri-implant fractures and the role of Short Proximal Femur Nails (SPFNs) in their management remains poorly addressed in the literature.

SPFNs have become a common treatment option for unstable extracapsular fractures due to their minimally invasive nature and mechanical advantages [3,4]. Nails are also becoming the preferred construct for stable fractures [5,6]. However, their use also presents unique complications, such as altered bone elasticity and stress concentrations, which can predispose them to peri-implant fractures [7,8]. The risk of fractures at the distal tip of the implant is over three times higher for intramedullary devices than for extramedullary implants [9]. Although peri-implant fractures can occur intraoperatively, they are more common between 6 and 10 weeks postoperatively or later. They are typically low-energy fractures resulting from falls from standing height, although they can also result from rotations of the operated limb or improper handling of the patient. These fractures are challenging due to their complex biomechanics, the difficulty in achieving stable fixation, and the potential for significant functional loss. Despite their clinical importance, there is limited research addressing these specific issues.

Given the increasing impact of femoral peri-implant fractures (PIFFs), classification systems are emerging, although agreement on this topic is still limited. The earliest classification by Duncan CP and Haddad FS in 2013 introduced the Unified Classification System (UCS) for both peri-implant and periprosthetic fractures, applicable to any long bone [10]. In 2018 and 2019, Chan et al. and Egol et al. published separate classifications for periprosthetic fractures but not specific to the femur [11,12]. Also in 2019, a Spanish team led by M Videla proposed a classification for PIFFs [8], based on the fracture location relative to the implant, whether at the implant end, through it, or away from it, using an alphanumeric code inspired by the AO/OTA and Vancouver classifications.

Management options include implant removal and a new osteosynthesis with longer implants, which carry risks such as greater surgical complexity and infection. Techniques like overlapping or interlocking implants may provide enhanced stability by protecting the entire femur, as emphasized in the 2024 Peri-Implant Spanish Consensus (PISCO) [13].

Despite the growing incidence of PIFs in aging populations, research gaps persist in understanding their risk factors, treatment options, and prevention strategies. This study aims to address these gaps by investigating the outcomes of the peri-implant hip fractures treated at our hospital, evaluating the potential causes of such fractures, determining the type of treatment provided, assessing the outcomes of said treatments, and establishing possible improvement strategies.

## 2. Materials and Methods

Following approval from the Ethics Committee (Ref. CEIm: 23-PI043), we conducted a retrospective observational analytical study that included all patients surgically treated for peri-implant hip fractures at the Orthopedic Surgery and Traumatology Department of Río Hortega University Hospital (HURH), a tertiary hospital, between 2010 and 2022.

The inclusion criteria for the study consisted of patients who underwent surgery for peri-implant hip fractures around a short cervicocephalic nail at the hospital facilities between 2010 and 2022 with a follow up of at least 12 months. Patients who sustained mechanical complications such as cut-out, cut-in, or cut-through in non-consolidated fractures were excluded, these being considered a complication of the osteosynthesis itself. These exclusion criteria were designed to avoid potential confounding and ensure that the study focused on true peri-implant fractures. A total of 33 consecutive patients were included, using their medical record numbers throughout the study period. The decision-making process for surgical strategies was structured based on fracture type, patient comorbidities, and implant characteristics. Long nails were preferred for diaphyseal fractures to enhance stability, while condylar plates were preferred for distal fractures to allow overlapping techniques. Implant selection also prioritized avoiding impingement on the anterior cortex and maintaining biomechanical integrity.

Patients were followed until the conclusion of the study in December 2023.

Data were obtained from the hospital’s electronic medical records. The data collected included the following: demographics (date of birth, sex), initial fracture characteristics (laterality, type of initial fracture, classification of the peri-implant fracture [8,14]), peri-implant fracture classification, implant details (type of implant, length, diameter, angulation, cephalic locking, Tip–Apex Distance [15], Cleveland quadrant [16], type of distal locking of the original nail), surgical outcomes (date of the first surgery, date of the peri-implant fracture, date of the peri-implant surgery, implant used in the second surgery, use of augmentation, surgical time, hospital stay, transfusions, presence and date of bone healing), functional scores (functional capacity before and after surgery using the Parker Mobility Score [PMS]), and complications (postoperative complications, mortality).

Patients or their families were also contacted by phone to assess functional capacity before and after surgery using the Parker Mobility Score (PMS). The PMS evaluates the patient’s ability to move indoors, on the street, and while shopping, scoring each activity on whether it can be done without difficulty (3 points), with an aid (2 points) or the assistance of another person (1 point), or not at all (0 points). The final score ranges from 0 to 9, with lower scores indicating a lower functional capacity [17].

### Classification

The fractures in our sample were classified according to the Videla classification, [8,14], which employs an alphanumeric code composed of two initial digits indicating the fracture location, followed by two or three letters identifying the type of fracture produced. The first two digits reference the AO/OTA classification, where the first digit indicates the femur (3) and the second indicates the proximal (1), diaphyseal (2), or distal (3) segment. For the subsequent characters (third position), the nomenclature used is similar to the Vancouver classification (utilized for peri-prosthetic fractures), in which “A” refers to fractures at the proximal end of the femur, “B” to short and oblique fractures at the end of the nail or involving the distal screw, and “C” represents fractures occurring distally to the nail. Additionally, the letter “N” denotes a nail and “P” refers to a plate. The fifth and final digit defines whether the implant was placed anterogradely (P) or retrogradely (D). This classification was tested by 35 traumatologists of varying experience, and no significant interobserver differences were found, concluding that it is a valid and reliable classification. To avoid any interobserver variability in fracture classification, all fractures were classified upon the agreement of two independent investigators.

All data were recorded in a Microsoft Excel spreadsheet (Microsoft Excel for Mac v16, Microsoft Corporation, Redmond, WA, USA), which was used to derive new variables such as time to surgery, time between the original fracture and peri-implant fracture, patient age at each fracture, age at death, and survival post-peri-implant fracture. This tool also facilitated graph generation and statistical calculations using SPSS Statistics (SPSS Statistics for Mac v27, IBM Corporation, Armonk, NY, USA) for further data analysis.

## 3. Results

There were 33 patients (91% women), with 34 peri-implant fractures (PIFs) and a mean age at the time of fracture of 87.6 years (standard deviation (SD) 6.2, range (R) 70 to 98 years).

In terms of the original fractures, the average age was 83.6 years (median 85, SD 6.2, R 65–97). The mean time between the initial fracture and the peri-implant fracture was 47.2 months (median 28, SD 41, range 0–194), with nine patients experiencing a fracture within the first 2 months post-surgery. The mean time from the occurrence of the PIF to surgery was 2.56 days (SD 1.13, R 0–7). The classification used to evaluate the periclavicular fractures under study was proposed by the Miquel Videla team: 13 (32-BNP), 8 (32-CNP), 6 (33-CNP), 5 (32-BND), 1 (31-ANP), and 1 (32-BNP + BPD).

**Figure 1:**
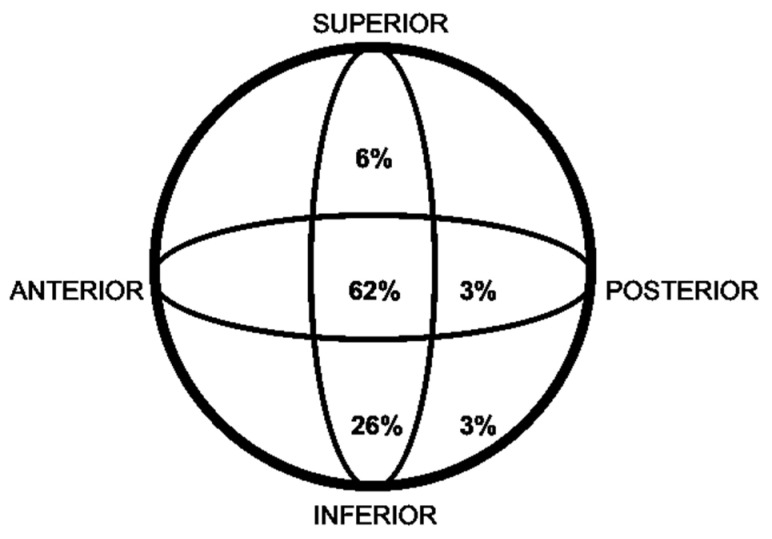
Position of the nail’s proximal screw/blade within the femoral head according to Cleveland and Bosworth quadrants.

The implants on which the fractures occurred were short intramedullary nails, of which 9 (27%) were TFN, 8 (24%) GAMMA, 14 (42%) PFNA, 1 (3%) AFFIXUS, and 1 (3%) ZNN. One patient who suffered a fracture on a GAMMA nail and was treated with a LISS plate experienced another PIF. The characteristics of the implants were as follows: we had nails ranging in length from 17 cm to 24 cm, with diameters ranging from 10 to 12 mm, and a cervicodiaphyseal angle of 130° in all cases, except for one AFFIXUS nail with an angle of 125°. We used 13 screws (38%) for cephalic locking and plates in 21 cases (62%). Their position in the femoral head was assessed according to Cleveland quadrants and Baumgaertner’s Tip–Apex Distance (TAD), achieving center–center and a TAD < 25 mm in 61.7% of cases (Figure 1).

The treatment of these fractures was performed through the extraction of the osteosynthesis material and the implantation of a new long nail in 26 (76%) cases, treating all 26 fractures (76%) affecting the diaphyseal segment of the femur (32-BNP, 32-CNP, and 32-BNP + BPD). Of these 26 fractures, 8 (31%) required cementation (five long PFNA^®^ nails and three long TFNA^®^ nails). There were seven fractures (six of type 33-CNP and one 32-CNP inter-implant) treated with a condylar plate osteosynthesis, in six of which the overlapping technique was performed, while in the one treated with a condylar plate that was not overlapped with the femoral nail, a third fracture (BNP + BPD) occurred 2 months later. The only fracture in the cervicotrochanteric region (type A) recorded was treated with a cemented partial hip prosthesis (Thompson).

The time elapsed from when the patient was admitted with the PIF until surgery was 2.56 days (SD 1.13, R 0–7). The mean duration of surgery was 102 min (SD 26.45, R 40–170), and the mean hospital stay was 9.6 days (median 8, SD 3.13, R 4–29). Thirty-one patients required transfusions during their stay, with an average of 2.65 units of red blood cells per patient (SD 1.17, R 0–8). Of the 33 patients, 24 (73%) did not present postoperative complications; 2 tested positive for SARS-COV-2, 1 had an infection of the plate, 1 had an atypical fracture, 1 presented a popliteal thrombosis, and 3 (9%) died during hospitalization. In 24 fractures (72%), radiological signs of consolidation were observed at 6.04 months (median 3, SD 4.76, R 1–36); eight patients died before consolidation (24%), one of whom refractured before consolidating, and another has not yet consolidated, presenting a delay in consolidation.

**Figure 2:**
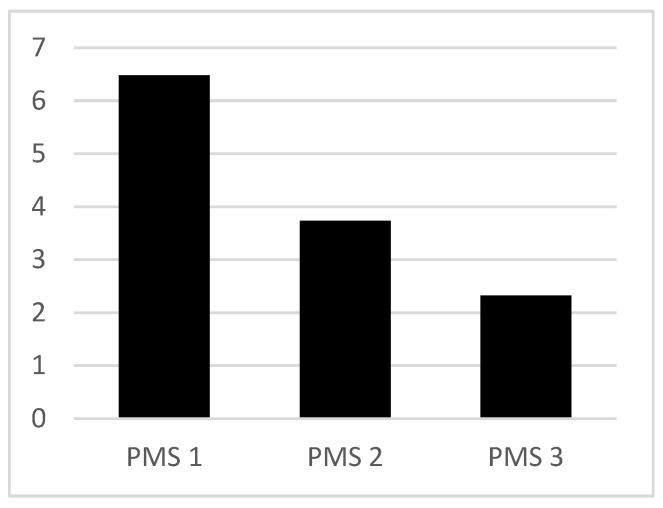
Evolution of functional capacity (PMS—Parker Mobility Score) before the index fracture (PMS1), after the index fracture (PMS2), and after the peri-implant fracture (PMS3).

Patients lost an average of 4.16 points in the Mobility Score (PMS) between the first hip fracture and the peri-implant fracture (64.19% of functionality). The loss of function due to the peri-implant fracture was 1.31 points (35.12%). The evolution of the average functional capacity of the patients is shown in Figure 2.

Analyzing functional capacity as it related to survival, we found a positive and statistically significant correlation for the Parker Mobility Score pre-index fracture (PMS1 correlation coefficient 0.354, *p* < 0.05), pre-peri-implant fracture (PMS2 correlation coefficient 0.647, *p* < 0.001), and post-peri-implant fracture (PMS3 correlation coefficient 0.604, *p* < 0.001).

**Figure 3:**
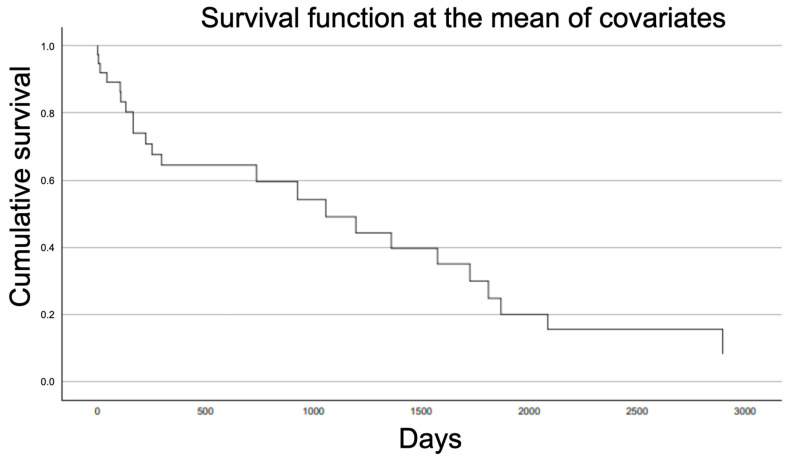
Kaplan–Meier curve of survivorship after PIF.

The overall survival of the patients can be observed through the Kaplan–Meier graph (Figure 3). The mortality at one year post-PIF was 36%, and at five years, it was 83%. No statistically significant differences were observed between the survival of fractures treated with the extraction of osteosynthesis material + a long intramedullary nail and those treated with condylar plate osteosynthesis (*p* = 0.75). Similarly, no statistically significant differences were observed between the BNP-type peri-implant fractures and those of type CNP (*p* = 0.124). The Pearson correlation coefficient (r) indicates a statistically significant negative correlation between the age at which the peri-implant fracture occurs and the patient’s survival (r (34) = −0.424, *p* < 0.05). Additionally, the Spearman correlation coefficient reveals a strong statistically significant correlation between the PMS and survivorship (Table 1).

## 4. Discussion

The literature surrounding peri-implant femoral fractures (PIFFs) is limited, primarily due to the absence of a standardized classification system. Nonetheless, several proposals have emerged in recent years to facilitate their understanding and management, including those by Chan et al. [11], Egol et al. [12], and Videla et al. [8,14]. However, due to the increasing incidence of these fractures, we anticipate that more studies like the present one will emerge. Most of the reviewed studies report low case numbers, hindering significant findings. The largest reported cohorts include those of Muller et al. [9], with 18 cases of PIFFs (15 of which were specifically sustained around a nail), and Kruse et al. [18], with 41 cases, of which only 3 were around a nail. Chan et al. [11] conducted the most extensive study to date in 2018, identifying 60 peri-implant fractures, 38 of which were femoral and only 3 around short femoral nails. Our study may represent one of the largest cohorts to date concerning the PIFFs of short cephalomedullary nails, comprising a total of 34 cases.

The true incidence of periprosthetic femoral fractures remains uncertain. In the 1990s, early intramedullary nail designs resulted in an incidence as high as 15% [19]. Currently, advancements in osteosynthesis systems for intertrochanteric fractures have reduced this incidence to approximately 1–2%, as evidenced by cohort studies with 1965 patients by Kruse et al. [18] reporting 0.8% and 1314 patients by Muller et al. [9] indicating 2.1%. Notably, the average time between the initial fracture and the occurrence of a peri-implant fracture in these studies was 27 and 23.6 months, respectively, while our study reported a longer interval of 47.2 months. This difference can be attributed to the high variability in follow-up duration; Muller et al. followed patients for an average of 12 years, and Kruse et al. for 9 years, whereas our retrospective study included patients whose initial fractures occurred over a decade ago. Despite this, our median follow-up period (28 months) aligns more closely with the existing literature. In any case, these findings highlight the importance of long-term advice for patients with femoral implants.

In terms of the mortality associated with PIFFs, Kruse et al. reported a 34% rate within the first year, while Muller et al. documented 23%, with our study yielding a mortality rate of 36%. The slight elevation in our findings may be explained by the aging population in Valladolid compared to other regions. Although these results align with previous studies, targeted interventions are needed to address the unique demographic and clinical challenges of our population.

Jennison et al. [20] analyzed 29 peri-implant fractures retrospectively in 2018 and found that 34% of patients ambulated independently before surgery (15% in our study), while 17% used a cane (15% in ours), and 17% relied on two canes (18% in ours). Furthermore, 31% used a walking frame (18% in our cohort, with an additional 35% unable to walk). These discrepancies may result from limited sample sizes and interobserver variability, as some wheelchair-bound patients may retain the ability to ambulate with assistance. Additionally, the average time to surgery in Jennison et al.’s study was 86.1 h (R 16–277) compared to 61 h in our study, while the average hospital stay was 13 days (R 6–144) versus 9.6 days (R 4–29) in ours. Our findings indicate that at our facilities, this type of fracture is managed with an increased priority and shorter hospital stays. Our shorter hospital stays and reduced surgical delays may reflect an improved prioritization of these fractures in our clinical setting, contributing to better functional outcomes despite a higher initial mortality.

A meta-analysis by Ma KL et al. [21] found no statistically significant differences in the incidence of PIFFs between PFNA^®^ nails and GAMMA^®^ nails. However, they concluded that the rate of refractures was higher with intramedullary nails than with sliding screw–plate devices (DHSs). In terms of surgical delays, a meta-analysis of 3000 periprosthetic fractures [22] revealed an average time from admission to surgery of 64 h, highlighting the significantly lower mortality rates associated with early surgical intervention compared to delayed procedures. Additionally, shorter hospital stays were linked to fewer medical complications, reduced transfusion needs, and a lower risk of reoperation, though the quality of the evidence was low. Our study reported an average surgical delay of 61 h; however, with 33 patients, we did not identify sufficient differences to establish a definitive conclusion, despite a weak non-significant association between mortality and surgical delay (Pearson correlation coefficient (r) = 0.26, *p* = 0.143).

The length of the intramedullary nail is another critical consideration. Numerous studies suggest that the risk of peri-implant fractures is comparable between short and long nails [23,24,25,26]. Cinque et al. [27] found no significant differences in the rate of peri-implant fractures between the two lengths, favoring short nails for pertrochanteric fracture treatment due to benefits such as a reduced blood loss, shorter surgery duration, and lower complication rates. Our study exclusively analyzed short cephalomedullary nails in initial fractures. Regarding distal locking, all patients in our cohort had distally locked intramedullary nails, with dynamic locking employed in the majority (88%). Skála-Rosenbaum et al. [28] concluded that distal locking helps prevent peri-implant fractures and recommended its use, while considering the possibility of non-locked intramedullary nails in certain stable pertrochanteric fractures, provided the nail adequately fills the intramedullary cavity. Notably, 15% of the peri-implant fractures in our study occurred right at the level of the distal locking screw, indicating a need for further evaluation. We believe there may be a relationship between these fractures and the force applied by the surgeon when tightening the distal locking screw, particularly given that, in most cases, the distal screw barely extended through the second femoral cortex. This positioning may act as a stress riser, creating a focal point of weakness in the femur.

The treatment of PIFFs continues to be a subject of debate, with various options available depending on the existing osteosynthesis material. The presence of prior implants may complicate reduction and obstruct the medullary canal, making the placement of new devices more challenging. One approach involves removing the original implant and performing a new osteosynthesis with a longer device. However, this entails a more extensive surgical approach, greater blood loss, and a higher risk of infection. Moreover, the mechanical strength of the bone decreases post implant removal due to underlying osteopenia and cortical loss at screw holes. Techniques such as overlapping old and new implants, including the use of femoral plates or interlocking both implants to create a stable construct covering the entire femur, may enhance stability and mitigate weak points prone to refractures. A recent consensus from a Spanish team (PISCO) [13] on the management of PIFFs emphasizes the importance of biological fixation, prioritizing closed reduction and minimally invasive techniques. It also advocates for the overlapping of implants or kissing techniques whenever feasible, conserving existing osteosynthesis implants, and protecting the femoral neck.

**Figure 4:**
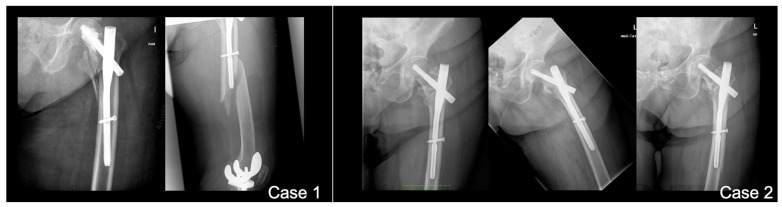
**Error #1—anterior cortical impingement.** Longer versions of short nails (exceeding 200 mm in length) can impinge on the anterior cortex of the femur, creating a stress concentration point at the tip of the nail that may lead to a fracture. To avoid this complication, the use of anatomically designed nails (available in left and right models) or long nails is recommended.

**Figure 5:**
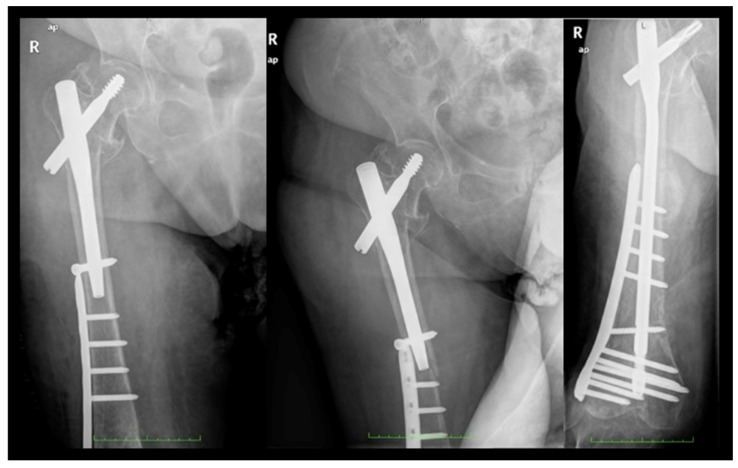
**Error #2—improper overlapping.** When a type C fracture is present, the original implant should be retained if possible to protect the femoral neck. In such cases, the use of a distal retrograde plate with overlapping is recommended. For enhanced protection, the overlapping should extend as proximally as possible. To improve fixation, various implant options are available, including periprosthetic plates, periprosthetic screws, variable-angle plates, and locking attachment plates, among others.

**Figure 6:**
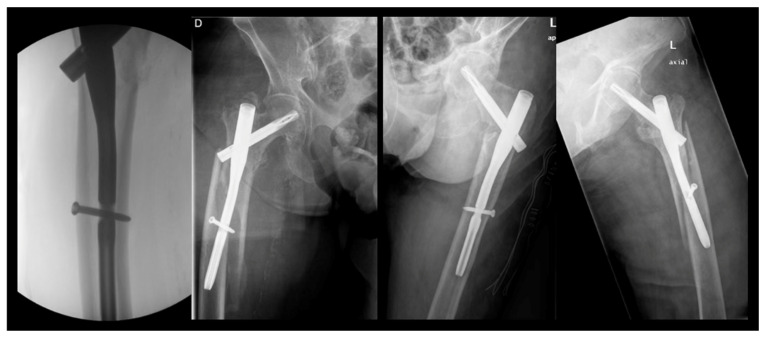
**Error #3—the overtightened screw.** The correct measurement of the distal locking screw is crucial. When the screw length is underestimated, the surgeon might overtighten it in an attempt to reach the second cortical of the femoral diaphysis. In such cases, the combination of the weakness created by the screw hole and the stress riser caused by the overtightened screw can lead to a fracture.

With 26.4% of patients experiencing fractures within 2 months post surgery of the index fracture, we advise a careful surgical technique to further improve outcomes and minimize the risk of PIFFs. The following strategies can be adopted. Surgeons should avoid overtightening distal locking screws against the outer cortical bone of the femur. Utilizing short femoral nails that do not impinge upon the anterior cortical bone (or considering the use of anatomical left and right nails) can also be beneficial. Employing overlapping techniques when other implants are present in the femur is advisable, while preventing the errors and outcomes presented in Figure 4, Figure 5 and Figure 6. Furthermore, using protective medications for bone fragility, such as anti-resorptives, following the diagnosis of an initial fracture (index fracture) may enhance patient outcomes.

This study provides valuable insights into peri-implant hip fractures, with strengths including its detailed analysis, practical surgical recommendations, and robust use of the Parker Mobility Score. However, its retrospective design, single-center setting, small sample size limiting subgroup analyses, and lack of long-term follow-up reduce its generalizability. Despite these limitations, it addresses important gaps and offers a practical framework for managing these complex fractures.

## 5. Conclusions

Peri-implant fractures (PIFs) in elderly patients are associated with high mortality rates and a considerable loss of functional mobility. A significant number of these fractures occur within the first two months post-surgery, emphasizing the need for meticulous surgical techniques to minimize the risk of early refractures. The observed decline in mobility after a PIF directly impacts patients’ quality of life and correlates with survival outcomes. It is important to enhance surgical approaches and post-operative management to support these frail patients and improve their overall prognosis.

## Figures and Tables

**Table 1:** Correlation coefficients (Spearman). There was a strong correlation between the Parker Mobility Score before and after the peri-implant fracture (PMS2 and PMS3) and survivorship.

	PMS1	PMS2	PMS3
Survivorship -Correlation coef -*p* value	0.354 0.04	0.647 <0.001	0.604 <0.001
PMS1 -Correlation coef -*p* value		0.583 <0.001	0.371 0.03
PMS2 -Correlation coef -*p* value			0.807 <0.001

## Data Availability

Data available on request due to restrictions.
